# Systemic and Tissue Inflammation in Juvenile Dermatomyositis: From Pathogenesis to the Quest for Monitoring Tools

**DOI:** 10.3389/fimmu.2018.02951

**Published:** 2018-12-18

**Authors:** Judith Wienke, Claire T. Deakin, Lucy R. Wedderburn, Femke van Wijk, Annet van Royen-Kerkhof

**Affiliations:** ^1^Laboratory of Translational Immunology, University Medical Center Utrecht, Utrecht, Netherlands; ^2^UCL Great Ormond Street Institute of Child Health, University College London, London, United Kingdom; ^3^NHR Biomedical Research Center at Great Ormond Hospital, London, United Kingdom; ^4^Arthritis Research UK Center for Adolescent Rheumatology, UCL, UCLH and GOSH, London, United Kingdom; ^5^Pediatric Rheumatology and Immunology, University Medical Center Utrecht, Utrecht, Netherlands

**Keywords:** juvenile dermatomyositis, tissue inflammation, vasculopathy, disease monitoring, biomarkers, interferon signature, autoantibodies, personalized medicine

## Abstract

Juvenile Dermatomyositis (JDM) is a systemic immune-mediated disease of childhood, characterized by muscle weakness, and a typical skin rash. Other organ systems and tissues such as the lungs, heart, and intestines can be involved, but may be under-evaluated. The inflammatory process in JDM is characterized by an interferon signature and infiltration of immune cells such as T cells and plasmacytoid dendritic cells into the affected tissues. Vasculopathy due to loss and dysfunction of endothelial cells as a result of the inflammation is thought to underlie the symptoms in most organs and tissues. JDM is a heterogeneous disease, and several disease phenotypes, each with a varying combination of affected tissues and organs, are linked to the presence of myositis autoantibodies. These autoantibodies have therefore been extensively studied as biomarkers for the disease phenotype and its associated prognosis. Next to identifying the JDM phenotype, monitoring of disease activity and disease-inflicted damage not only in muscle and skin, but also in other organs and tissues, is an important part of clinical follow-up, as these are key determinants for the long-term outcomes of patients. Various monitoring tools are currently available, among which clinical assessment, histopathological investigation of muscle and skin biopsies, and laboratory testing of blood for specific biomarkers. These investigations also give novel insights into the underlying immunological processes that drive inflammation in JDM and suggest a strong link between the interferon signature and vasculopathy. New tools are being developed in the quest for minimally invasive, but sensitive and specific diagnostic methods that correlate well with clinical symptoms or reflect local, low-grade inflammation. In this review we will discuss the types of (extra)muscular tissue inflammation in JDM and their relation to vasculopathic changes, critically assess the available diagnostic methods including myositis autoantibodies and newly identified biomarkers, and reflect on the immunopathogenic implications of identified markers.

## Introduction

Juvenile Dermatomyositis (JDM) is a systemic immune-mediated disease of childhood. It is the most common idiopathic inflammatory myopathy in children, with an incidence of 2–4/million/year (1). Although the exact etiology is still elusive, both genetic and environmental factors are thought to play a role in the development of the disease (2–5). JDM is characterized by inflammation of skeletal muscles and skin, leading to muscle weakness and a typical skin rash of the face and hands (heliotrope rash and Gottron's papules, respectively), which are also used as classification criteria (6, 7). Next to the muscle and skin, other organs can be affected. Vital organ involvement, especially of the lungs, is still the major cause of death in JDM patients (8, 9). Although rare, cardiac involvement and microangiopathy of the intestine, brain and kidneys have been described (10). Thus, rather than being confined to specific tissues, JDM is a truly systemic disease, which can affect multiple organ systems.

Before the introduction of corticosteroids as a treatment option, mortality and morbidity among JDM patients were high, and long-term outcomes were not the primary focus. Since then, mortality rates have dropped from over 30% to 2–3% (11). With increasing survival, long-term outcomes become an important concern of patients and physicians, as patients' quality of life and societal participation depend on it. Long-term outcomes are likely dependent on various factors such as disease severity and activity, response to treatment and medication side effects which together determine the cumulative organ and tissue damage.

Especially low-grade inflammation and extramuscular manifestations of the disease are difficult to investigate in routine clinical care and may therefore be overlooked. Unrecognized, local inflammation leading to tissue damage and subsequent organ dysfunction may have serious consequences for short-term and long-term outcomes. So far, reliable assessment of disease activity and the type and extent of tissue involvement has been rather challenging. Current clinical tools for assessment of disease activity require active collaboration of patients, which can be difficult for young, unwell children. Detecting low-grade inflammation or differentiating clinically between various causes of muscle impairment is even more challenging. Hence, there is a great need for minimally invasive, objective and reliable diagnostic tools for the assessment and monitoring of (low-grade) disease activity and related organ involvement. Optimally, such tools could guide clinical decision making, facilitate individually tailored treatment regimens, and reduce the risk of over- and under-treatment.

In this review we will discuss the types of (extra)muscular tissue involvement that have been described in JDM and their relation to vasculopathic changes, critically assess the available diagnostic and monitoring tools and reflect on the immunopathogenic implications of identified markers.

## Signs of Systemic Disease Activity in JDM Based on affected Tissues and Organs

JDM patients can present with a spectrum of symptoms. Most, but not all patients, have the classic combination of muscle involvement and typical skin rashes. Approximately 1–5% of JDM patients present with amyopathic JDM, but it was estimated that 26% of these patients will eventually progress to classical JDM, which can occur up to years after onset (12). This indicates that the phenotype can evolve over the course of the disease, possibly also dependent on treatment. True amyopathic JDM however is very rare and mild muscle involvement may be present but missed (13). Amyopathic JDM generally has a relatively mild disease course with fewer systemic manifestations, less required immunosuppressive treatment and a good prognosis (12, 14, 15).

### (Sub)Cutaneous and Other Extramuscular Symptoms

Cutaneous symptoms can range from the pathognomonic heliotrope rash and Gottron's papules, to photosensitive rashes such as malar and truncal erythema, and severe complications such as skin ulceration and dystrophic calcinosis. Calcinosis occurs in 12–47% of patients and can occur in the skin and in subcutaneous, myofascial, or muscle tissue. Most often it is a long-term complication and its presence has been associated with delayed diagnosis and more severe disease with poorer functional outcomes. Effective treatment of calcinosis is still challenging, but aggressive high-dose immunosuppression or, in very severe cases, autologous stem cell transplantation have been shown to be able to reverse calcinosis, suggesting that chronic (low-grade) inflammation may be accountable for calcifications (16–20). Cutaneous and oral ulceration affects up to 30% of patients and is thought to result from occlusive endartheropathy of the small vessels (10, 21). Lipodystrophy affects 8–14% of JDM patients and is often associated with hormonal and metabolic changes (10, 22–24). We suspect that patients with lipodystrophy may therefore have an increased risk of cardiovascular events in the long-term. Limb edema and arthritis are also common, occurring in 11–32 and 23–58% of patients, respectively (10).

Next to the skin and musculoskeletal system, other organ systems can be involved, of which the lung is the most frequently affected. Up to 75% of children with JDM develop respiratory involvement, which may result from a complication of respiratory muscle weakness or immunosuppressive therapy, or from interstitial lung disease (ILD) (25, 26). ILD occurs in 8–19% of juvenile myositis patients and has been described as the major cause of death in JDM (27–30). Cardiac involvement may be present subclinically more often than recognized, as even in JDM patients without clinical cardiac dysfunction abnormal ECG and echocardiographic findings are relatively common (31–33). Conduction abnormalities and myocarditis have been reported, and systolic and diastolic dysfunction was found after long-term follow-up (34–37). Cardiac complications are thought to result from myocarditis and coronary artery disease as well as involvement of the small vessels of the myocardium (38). Involvement of the gut or neural system are rare complications of JDM and are also thought to result from an underlying small vessel angiopathy or vasculitis (39–41). Intestinal consequences of the small vessel angiopathy include ulceration, perforation, hemorrhage, pneumatosis intestinalis and malabsorption (42–44).

### Vasculopathy

The pathologic changes underlying symptoms and tissue damage in the skin, muscles, and vital organs have a common factor: in all the affected tissues typical vasculopathic changes are observed, which include loss of capillaries (capillary dropout), perivascular inflammation, and (occlusive) small vessel angiopathy (21, 45). In a recently reported French JDM cohort of 116 patients, vasculopathy-related complications were the main cause of admission to the intensive care unit, illustrating the severity and relevance of vascular involvement in JDM (46). These complications include life-threatening disorders like systemic capillary leak syndrome, recently also described in 3 patients with JDM (47).

Deposition of complement, immune complexes and anti-endothelial antibodies is thought to play an important role in endothelial damage and subsequent capillary dropout (48–54). Clinically, the severity of vasculopathy and the disease phenotype have also been linked. The presence of prominent vascular injury in muscle biopsies identified a subgroup of patients with more severe clinical presentation and outcomes, including profound muscle weakness, limb edema and gastrointestinal involvement (55). This suggests that local vasculopathic changes can reflect systemic vasculopathy and the resulting clinical symptoms. Nailfold capillaroscopy, a commonly and easily used indicator of disease activity in clinical practice, is also based on this principle. The pathologic changes observed in nailfold capillaries, such as capillary dropout, branching and dilatation, likely reflect the systemic blood vessel abnormalities. Loss of end row nailfold capillaries is significantly associated with clinical disease activity scores for muscle and skin and can thus be used as a marker of skin and muscle activity. Nailfold capillaroscopy is especially suited as a non-invasive tool to follow up changes in disease activity over time in patients (56–59).

Taken together, JDM is a truly systemic disease in which not only the muscles and skin are affected, but also vital organs can be involved. The presence of typical vasculopathic changes in the various affected tissues points toward a central role for systemic endothelial dysfunction in the pathogenesis of JDM.

## Monitoring of Disease Activity and Tissue Involvement

During clinical follow-up, monitoring of disease activity is crucial to determine the rate of medication tapering or to assess the requirement for intensification of immunosuppressive therapy. Next to clinical evaluation, various tools have been investigated for monitoring of disease activity, among which autoantibodies and other circulating biomarkers, and histopathologic evaluation of muscle biopsies, as well as several imaging techniques.

### Clinical Assessment

The primary and most important evaluation of disease activity involves clinical assessment by experienced clinicians and health care professionals. Over the past years, several scoring tools have been devised for internationally standardized evaluation of disease activity (60). The most commonly used tools are now the childhood myositis assessment scale (CMAS), manual muscle testing of 8 muscle groups (MMT-8), physician's and patient's global assessment on a visual analog scale (PGA), cutaneous assessment tool (CAT), cutaneous dermatomyositis disease area and severity index (CDASI), disease activity score (DAS), myositis disease activity assessment tool (MDAAT) and childhood health assessment questionnaire (CHAQ) (61–69). Combined scoring systems are currently being developed (70). The Pediatric Rheumatology International Trials Organization (PRINTO) has composed criteria for defining clinically inactive disease (71). A recent re-evaluation of these PRINTO criteria showed that skin disease may be underestimated as a factor in the assessment of disease activity (72).

Clinical measures of disease activity, however, have limited capacity to detect low-grade inflammation in the tissues which does not cause overt symptoms, but may still contribute to tissue damage in the long term. Moreover, it is challenging to differentiate between various underlying causes of symptoms by clinical assessment. For example, muscle weakness may result from an ongoing inflammatory process, from medication side effects (e.g., steroid myopathy), muscle damage or effects of immobility. Biological assessment of the affected tissues and organs can therefore be helpful or even necessary to aid clinical decision-making concerning medication dose and additional interventions.

### Biomarkers for Disease Course, Activity, and Tissue Involvement

Laboratory investigation of blood is a minimally invasive and time-efficient procedure, especially compared to muscle biopsy and some of the imaging methods. It is therefore particularly suited as a method for serial sampling during clinical follow-up. Laboratory investigation can be used for measurement of autoantibodies and for biomarkers related to disease activity and specific (extra)muscular symptoms.

#### Autoantibodies

Antibodies found in myositis include myositis-specific autoantibodies (MSA), relatively specific to myositis, and myositis-associated antibodies (MAA), which are observed both in myositis and other connective tissue diseases (6). In the past years, different disease phenotypes have been linked to the presence of autoantibodies and particularly myositis-specific autoantibodies (16). The frequencies of autoantibodies in juvenile patients differ substantially from adult DM patients (73). Anti-TIF1 (p155/140) and anti-NXP2 (p140 or MJ) are the most commonly identified autoantibodies in Caucasian JDM patients (20–35 and 16–23%, respectively) (28, 73–76). Anti-TIF1 is associated with skin ulceration, photosensitive skin rashes, lipodystrophy, and edema (24, 75–78), whereas anti-NXP2 is associated with a severe disease course with more profound muscle involvement, calcinosis, gastrointestinal ulceration, joint contractures, and dysphonia (75, 77, 79, 80). A recently identified myositis specific autoantibody which is especially frequent in the Asian JDM population, is anti-MDA5 (CADM-140) (81). It is found in 33% of Asian JDM patients, compared to 7% of Caucasian patients (8, 82). Patients with anti-MDA5 have a higher risk of developing ILD than patients without these antibodies. This anti-MDA5 conferred risk is seen in both Asian and Caucasian JDM cohorts, although the risk difference appears to be more pronounced in Asian cohorts (8, 83). Common symptoms in Caucasian patients with anti-MDA5 antibodies include oral and cutaneous ulceration, arthritis, and milder muscle disease with fewer histologic abnormalities and a higher remission rate off medication after 2 years of follow-up (76, 82, 84, 85). Less frequently identified autoantibodies in the juvenile population include anti-Mi2 (4–10%) and anti-amino-acyl-tRNA synthetase antibodies such as anti-Jo-1 (1–3%) and anti-SAE (<1%). Anti-SRP and anti-HMG-CoA-reductase (Anti-HMGCR) autoantibodies, both accounting for <3% of juvenile myositis patients, are associated with a necrotizing type of myopathy with severe muscle weakness (73, 76, 86, 87).

It remains unclear whether each MSA reflects a distinct pathologic process, influencing the type and severity of disease phenotype and tissue involvement. Notably, autoantibodies against Jo-1, TIF1, SRP, and Mi-2 are not only informative at disease onset, but their levels have been found to correlate with disease activity during follow-up in the context of rituximab treatment (88). This highlights that perhaps autoantibodies should be measured during or soon after the first clinic visit as their levels may decline and become undetectable in remission.

A last and different (not myositis-specific) category of autoantibodies identified in JDM comprises autoantibodies against components of endothelial cells, which are thought to contribute to capillary loss. These anti-endothelial cell autoantibodies (AECA) were detected in 76% of JDM patients, as opposed to 30% of control patients (49). Twenty-two candidate target autoantigens for AECA were identified in JDM plasma, 17 of which were proteins associated with antigen processing and protein trafficking (50). Identification of autoantibody targets may provide novel insights into the auto-immune process and self-antigens involved in JDM.

#### Biomarkers for Systemic Inflammation and Muscle Disease Activity

Reliable assessment of disease activity during follow-up can be aided by laboratory markers that represent systemic and/or local inflammation. Especially for detection of low-grade inflammation and for differentiation between various causes of muscle weakness, laboratory investigation can be a helpful or even necessary tool.

So far, reliable and validated laboratory markers for disease activity and tissue involvement in JDM are still lacking. A large number of proteins in plasma, serum, and urine as well as circulating immune cell subsets have been investigated as potential biomarkers for (tissue-specific) disease activity in patients with JDM (Tables 1, 2). In theory, every biological parameter that can be measured, could serve as a biomarker. To be suited for use in clinical practice however, a biomarker has to meet additional criteria, such as being reliable, robust, relatively stable and easy to measure. In the following paragraphs we highlight all biological markers that have been associated with disease activity in JDM, regardless of their suitability for use in clinical practice, as some of these identified markers may still contribute to the understanding of the immunopathogenesis of JDM. However, it is important to note that due to the rarity of the disease, many of these studies were carried out in small cohorts of <30 patients (as outlined in Tables 1, 2). Insights based on such small numbers have limitations in a heterogeneous disease like JDM. Therefore, validation of identified markers in larger cohorts is crucial before implementation into clinical practice.

**Table 1:** Biomarkers for disease activity in JDM cohorts.

**Biomarker**	**Global disease activity**	**Muscle disease activity**	**Other activity measures**	**Patients**	**Material & technique**	**Cohort**	**References**
**Interferon related biomarkers**							
MxA		++++^**^ (DAS, O), +++^*^ (DAS, FU)	NS (skin DAS)	14 act JDM: 7 untreated, 7 treated	PBMC, qRT-PCR	USA	O'Connor 89
IFNα activity	###^*^ (DAS, off therapy at 36 months)	NS (DAS)	###^**^ (skin DAS, off therapy at 36 months), ^**^ (vsHC)	39 JDM and 19 ped HC	Serum, Functional reporter assay	USA	Niewold 90
IFN gene score	r_p_: NS (DAS)	r_p_: NS (DAS)		27 JDM	Whole blood, qRT-PCR	USA	Baechler 91
IFN chemokine score	r_p_: ++^**^ (DAS)	r_p_: ++^*^ (DAS)		29 JDM			
Eotaxin	+^**^ (DAS)	NS (DAS)	+^**^ (skin DAS), ^*^ (vsHC)	54 JDM	Serum, Luminex	Norway	Sanner 92
MCP-1	+ ^*^ (DAS)	NS (DAS)	NS (skin DAS), ^**^ (vsHC)	54 age+sex matched controls			
IP-10	NS (DAS)	NS (DAS)	NS (skin DAS), ^*^ (vsHC)	Median time 16.8 yrs after onset			
IP-10	+++^***^ (PGA)	###^***^ (CMAS)		2014: 25 JDM (18 act, 19 rem), 14 ped HC, 8 NIMD	Plasma and serum, Luminex	NL	Enders 93, 94
TNFR2	+++^***^ (PGA)	NS (CMAS)					
Galectin-9	+++^***^ (PGA)	##^**^ (CMAS)		2015: 3 refractory JDM (pre and post aSCT)			
Soluble IL-2R	^**^ (diagnosis vs. rem)			7 JDM: 7 at diagnosis, 7 in rem	Serum, ELISA & HPLC	Japan	Kobayashi 95
Neopterin	Higher in act than rem						
Neopterin		+++^***^ (act), ++++^*^ (FU 3 pts) (both with study-specific DAS)		15 JDM (21 samples: 12 act, 9 rem)	Serum, radioimmunoassay	Italy	De Benedetti 96
Urine neopterin	++^**^ (PGA)	###^*^ (MMT), ##^**^ (CMAS)	++^**^ (skin VAS), ++^**^ (CHAQ), ++^**^ (MRI)	39 JDM, 3 JDM with overlap CTD, 3 JPM	Urine and plasma, ELISA, HPLC, gas chromatographic mass spectrometry	USA	Rider 97
Urine quinolonic acid	++^**^ (PGA)	###^*^ (MMT), ##^***^ (CMAS)	++^***^ (CHAQ), ++^**^ (MRI)				
Plasma neopterin/quinolonic acid	NS (PGA)	NS (CMAS, MMT)					
**OTHER MARKERS OF INFLAMMATION**							
MRP8/14	+++^***^ (PGA)	##^**^ (CMAS)	NS (CHAQ)	56 JDM	Serum, ELISA	UK	Nistala 98
CRP			Low during relapse in 4 patients	9 JDM: 4 during relapse and 3 rem	Serum	UK	Haas 99
**IMMUNE CELL SUBSETS**							
Changes in %CD19+ cells	++^*^ (DAS)				PBMC, flow cytometry	USA	Eisenstein 100
T cell subsets	NS (DAS)						
T cell activation (CD25, HLA-DR)	NS (DAS)						
T cell recognition of human Hsp60	Higher in rem than act			22 JDM: 6 new-onset, 6 act, 10 rem	PBMC, ^3^H-thymidine assay	NL	Elst 101
Th1 within CXCR5+ CD4 T cells	Higher in rem than act^***^			45 JDM (52 samples): 26 act, 26 rem, 43 ped HC	PBMC, Flow cytometry	USA	Morita 102
Ratio (Th2+Th17)/Th1 in CXCR5+ CD4 T cells	Higher in act than rem^***^						
% Plasmablasts (CD19+CD20–CD27+CD38++)	Higher in act than rem^***^						
Change in % CD3+CD69+ T cells	++^*^			24 JDM	PBMC, Flow cytometry	USA	Ernste 103
Change in HLA-DR- CD11c+ mDC				++^*^ (extra)			
Change in HLA-DR- CD123+ pDC		##^*^					
% FOXP3+ Tregs		NS (CMAS)		48 JDM: 21 act, 27 rem	Muscle biopsies, immunohistochemistry	NL	Vercoulen 104
Defective suppressive function of Tregs	In 4/11 active pts vs. 0/9 in remission					Flow cytometry, ^3^H-thymidine incorporation	
RORC	^*^	^*^	NS (extra)	26 JDM new-onset	Whole blood, qRT-PCR	USA	Lopez de Padilla 105
IL-17F	NS	^*^	NS (extra)				
GATA3	NS	^*^	NS (extra)				
STAT4	^***^	^***^	NS (extra)				
Changes in STAT6	NS	NS	^*^ (extra)				
Changes in IL-17D	NS	NS	^**^ (extra)				
Changes in BCL6	NS	NS	^**^ (extra)				
% Immature transitional B cells	+++^***^ (PGA)			68 JDM (113 samples): 20 pre-treatment, 93 on treatment	PBMC, Flow cytometry	UK	Piper 106
Absolute number immature transitional B cells	+++^***^ (PGA)						
**MARKERS RELATED TO ENDOTHELIAL ACTIVATION OR DYSFUNCTION**							
vWF			Sens 0.85; Spec 0.45 (for flare)	16 JDM, prospective	Serum	CA	Guzman 107
vWF	Sens 0.40 (6/15 act had high vWF)	NS (muscle strength)	NS (skin rash, calcinosis)	15 JDM	Serum	USA	Bloom 108
C3d	Elevated in 6/7 pts with act			15 JDM: 7 act, 5 mild disease, 3 rem, 15 ped HC	Plasma, radioimmuno assayand rocket immuno-elektrophoresis	USA	Scott 109
Fibrinopeptide A			^*^ (vsHC)				
Factor VIII-related antigen			^**^ (vsHC)				
MiRNA-10a	NS (DAS)	NS (DAS)	NS (skin DAS)	15 untreated JDM	Muscle boipsies, RT-PCR	USA	Xu 110
EPC number		NS (DAS)	NS (skin DAS)	34 JDM: 6 untreated, 19 act on med, 9 rem	PBMC, Flow cytometry	USA	Xu 111
**LIPID METABOLISM**							
HDL	NS (PGA)	++^*^ (CMAS), NS (MMT)	NS (Skin, CHAQ)	16 JDM, 1 JPM	Serum	USA	Coyle 112
LDL	r_p_: ++^*^ (DAS)	r_p_: ##^*^ (CMAS)	r_p_: ++^**^ (MYOACT)	25 JDM	Serum	Brazil	Kozu 113
Triglycerides	All r_p_: +++^**^ (DAS), ++^**^ (MITAX)	All r_p_: ##^**^ (CMAS), ##^**^(MMT)	r_p_: +++^**^ (MYOACT)				
IL-6	NS	#^*^	NS (extra)	26 JDM	Whole blood, qRT-PCR	USA	Olazagasti 114
Resistin	++^***^	++^***^	+^*^ (extra)				

Spearman correlations (r_s_) of biomarkers with global/muscle/skin/extraskeletal VAS are shown unless otherwise specified. +:r_s_ ≥0.2, ++:r_s_ ≥0.4, +++:r_s_ ≥0.6, ++++:r_s_ ≥0.8, #:r_s_ ≤ −0.2, ##:r_s_ ≤ −0.4, ###:r_s_ ≤ −0.6, ####: r_s_ ≤ −0.8. r_p_: Pearson correlation. Statistical significance:

* *P < 0.05*,

** *P < 0.01*,

*** *P < 0.001, NS, not significant. Sens, sensitivity; Spec, specificity. Abbreviations biomarkers: IFN, interferon; MCP-1, CCL2; IP-10, CXCL10; TNFR2, Tumor necrosis factor receptor 2; IL-2R, Interleukin-2 receptor; MRP8/14, myeloid related protein 8/14 (S100A8/9); CRP, C-reactive protein; Hsp60, heat shock protein 60; Th, T helper; mDC, myeloid dendritic cell; pDC, plasmacytoid dendritic cell; Treg, Regulatory T cell; IL, interleukin; vWF, von Willebrand factor; EPC, endothelial progenitor cell; HDL, high density lipoprotein; 25(OH)D, Vitamin D. Abbreviations disease activity: DAS, disease activity score; VAS, visual analog scale; PGA, physician's global activity VAS; MyoAct, Myositis disease activity assessment visual analog scales; MITAX, myositis intention to treat activity index; MMT, manual muscle testing; CMAS, childhood myositis assessment scale; vsHC, compared to healthy controls; CHAQ, childhood healthy assessment questionnaire; MRI, magnetic resonance imaging; ANA, anti-nuclear antibody; extra, extraskeletal/extramuscular symptoms. Abbreviations patients: JDM, juvenile dermatomyositis; JPM, juvenile polymyositis; HC, healthy control; ped, pediatric; act, active; rem, remission/asymptomatic/inactive disease; yrs, years; NIMD, non-inflammatory muscle disease; aSCT, autologous stem cell transplantation; CTD, connective tissue disease; O, onset of disease; FU, follow-up. Abbreviations material & technique: ELISA, enzyme-linked immuno sorbent assay; HPLC, high-performance liquid chromatography; PBMC, peripheral blood mononuclear cells; qRT-PCR, quantitative real time polymerase chain reaction*.

**Table 2:** Biomarkers for disease activity in mixed cohorts containing patients with JDM.

**Biomarker**	**Global disease activity**	**Muscle disease activity**	**Other activity measures**	**Patients (samples)**	**Material & technique**	**Cohort**	**References**
**Interferon related biomarkers**							
Type I IFN signature	NS			2 JDM, 10 DM, 15 ad HC	Whole blood, microarray	USA	Baechler 115
IP-10	NS					Serum, Luminex	
ITAC	NS						
MCP-1	++^*^						
MCP-2	+++^**^						
IFN gene score	++^**^	++^**^ (VAS), ##^**^ (MMT)	++^**^ (skin), +^*^ (extra)	19 JDM, 37 DM, 20 ad HC	Whole blood, qRT-PCR	USA	Bilgic 116
IP-10	+++^****^				Serum, multiplexed sandwich immunoassay		
I-TAC	+++^****^						
MCP-1	++^***^						
MCP-2	++^***^						
MIP-1α	++^***^						
IL-6	++^***^	+^**^ (VAS), ##^**^ (MMT)	+^*^ (skin), ++^**^ (extra)				
IL-10	+^*^						
TNFα	+^*^						
TNFR1	+^*^						
*MIG, MIP-1β, IL-8*	NS						
Type I IFN chemokine score (Summarization of ITAC, IP-10, MCP-1, and MCP-2)	++^*^ (JDM), +++^****^ (mixed)	++^***^ (VAS, mixed), ##^**^ (MMT, mixed)	+^*^ (skin), ++^**^ (extra)				
Type I IFN gene score	+^*^	++^**^	NS (extra)	21 JDM (each active and remission sample), 30 DM (each active and remission sample)	Whole blood, qRT-PCR	USA	Reed 117
Type I IFN chemokine score	++^***^	++^***^	++^***^ (extra)		Serum, multiplexed sandwich immunoassay		
IP-10	++^***^	++^**^	++^***^ (extra)				
ITAC	++^**^	++^**^	++^**^ (extra)				
MCP-1	++^**^	++^**^	++^**^ (extra)				
MCP-2	NS	++^***^	NS (extra)				
IL-6	++^***^	++^***^	+^*^ (extra)				
IL-8	+^*^	+^*^	NS (extra)				
TNFα	+^**^	++^***^	+^*^ (extra)				
**OTHER MARKERS OF INFLAMMATION**							
IL-1Ra	High in act than rem	++^*^ (CK)	^***^ (vsHC)	2 JDM, 5 DM, 2 caDM, 4 PM, 2 OM, 12 HC	Serum, ELISA	Switzerland	Gabay 118
sTNFR75 (sTNFR2)		++^*^ (CK)					
BAFF		+^***^ (CK, mixed)		49 DM (of which ≥1 JDM), 44 PM, 6 IBM, 30 HC	Serum, ELISA	Sweden & Czech Republic	Krystufkova 119
BAFF	+^***^ (mixed)	+^*^ (mixed)	+^***^ (extra, mixed)	20 JDM, 45 DM, 26 PM, 7 IBM, 21 HC	PBMC, qRT-PCR	USA	Lopez de Padilla 120
ΔBAFF (downregulates BAFF activity)	++^***^ (mixed)	+^**^ (mixed)	++^***^ (extra, mixed)				
Anti-Jo1	LMM: ++^***^	LMM: ##^***^ (MMT), +++^***^ (ME)	LMM: +^**^ (extra), ++^***^ (HAQ)	Refractory pts: 48 JDM, 76 DM, 76 PM (all analyses in mixed cohort)	Serum, ELISA & RNA and protein immunoprecipitation	USA	Aggarwal 88
Anti-TIF1γ	LMM: ++^**^	LMM: ###^***^ (MMT), NS (ME)	LMM: +++^***^ (HAQ), NS (extra)				
Anti-SRP	LMM: NS	LMM: NS (MMT), +^**^ (ME)	LMM: NS (extra, HAQ)				
Anti-Mi2	LMM: +++^***^	LMM: ##^***^ (MMT), ++^**^ (ME)	LMM: +^*^ (extra), NS (HAQ)				
**IMMUNE CELL SUBSETS**							
% CD3+ cells	Higher in rem than act^*^ (DM, not JDM)			14 JDM, 24 DM, 17 ad HC, 9 ped HC	PBMC, Flow cytometry	Japan	Ishida 121
% CD8+ cells	Higher in rem than act^*^ (DM, not JDM)						
% CD20+ cells	Higher in act than rem^*^ (DM, not JDM)						
% CD3+ cells	Higher in rem than act^*^ (DM)			29 DM act (of which ≥1 JDM), 20 DM rem, 13 PM act, 37 PM rem, 32 ad HC	PBMC, Flow cytometry	Hungary	Aleksza 122
% CD8+ cells	Higher in rem than act^*^ (DM)						
% IFNγ+ of CD4 T cells	Higher in rem than act^**^ (DM)						
% IFNγ+ of CD8 T cells	Higher in rem than act^**^ (DM)						
% CD19+ cells	Higher in act than rem^*^ (DM)						
% IL-4+ of CD4+ T cells	Higher in act than rem^*^ (DM)						
**MARKERS RELATED TO ENDOTHELIAL ACTIVATION OR DYSFUNCTION**							
sVCAM-1		NS (CK, mixed)	NS (skin, mixed)	5 JDM, 27 DM, 4 PM, 25 HC	Serum, ELISA	Japan	Kubo 123
sE-selectin			^*^ (CK, mixed)				
sICAM	Higher in act than rem^*^ (mixed)			4 JDM, 2 ped MCTD, 8 ped SLE, 4 ped Vasculitis	Serum, ELISA	USA	Bloom 124
sICAM-3, sVCAM-1, sL-selectin, sE-selectin	NS (mixed)						

Spearman correlations (r_s_) of biomarkers with global/muscle/skin/extraskeletal VAS are shown unless otherwise specified. +:r_s_ ≥ 0.2, ++:r_s_ ≥ 0.4, +++:r_s_ ≥ 0.6, ++++:r_s_ ≥ 0.8, #:r_s_ ≤ −0.2, ##:r_s_ ≤ −0.4, ###:r_s_ ≤ −0.6, ####:r_s_ ≤ −0.8. r:_p_: Pearson correlation, LMM, linear mixed model. Statistical significance:

* *P < 0.05*,

** *P < 0.01*,

*** *P < 0.001*,

**** *P < 0.0001, NS, not significant. Abbreviations biomarkers: IFN, interferon; MCP-1, CCL2; MCP-2, CCL8; IP-10, CXCL10; ITAC, CXCL11; MIP-1α, CCL3; IL, interleukin; TNF, Tumor necrosis factor; TNFR1 /2 = TNF receptor 1/2, MIG, CXCL9; MIP-1β, CCL4; IL-1Ra, IL-1 receptor alpha; BAFF, B cell activating factor. Abbreviations disease activity: CK, creatine kinase; ME, muscle enzymes; VAS, visual analog scale; MMT, manual muscle testing; HAQ, health assessment questionnaire; extra, extraskeletal/extramuscular symptoms. Abbreviations patients: JDM juvenile dermatomyositis; JPM, juvenile polymyositis; DM, adult dermatomyositis; caDM, cancer-associated DM; PM, adult polymyositis; OM, overlap myositis; IBM, inclusion body myositis; SPA, spondylartropathy; SLE, systemic lupus erythematosus; SSc, systemic sclerosis; MCTD, mixed connective tissue disease; HC, healthy control; ped, pediatric; ad, adult; act, active; rem, remission/asymptomatic/inactive disease. Abbreviations material & technique: ELISA, enzyme-linked immuno sorbent assay; PBMC, peripheral blood mononuclear cells; qRT-PCR, quantitative real time polymerase chain reaction*.

##### Currently used laboratory markers

The markers that are currently used in clinical practice, AST, ALT, LDH, aldolase and in particular creatine kinase activity (CK), do not correlate as well with disease activity in JDM as in DM (125–127). At diagnosis, any one muscle enzyme was only elevated in 80–86% of patients with JDM and CK was found to be elevated in only 61–64% of patients (125, 128). In almost 20% of patients the most abnormal measurement of CK was not elevated above normal values (28). Low muscle enzymes at first presentation may be associated with delayed diagnosis (129). During follow-up, CK may underestimate disease activity due to suppressed release by corticosteroids, circulating inhibitors of CK activity, or loss of muscle mass (127, 130–132). On the other hand, CK and aldolase can be elevated in steroid myopathy and are therefore not reliable as markers for disease activity requiring more potent immunosuppression (133). However, according to recent consensus guidelines, these muscle enzymes are still regarded as an important monitoring tool (134, 135).

##### Markers related to the interferon signature

An important group of investigated biomarkers is related to the type 1 interferon (IFN) signature, which has been demonstrated in the peripheral blood and muscle biopsies of JDM patients (136, 137). Activated plasmacytoid dendritic cells (pDC) are generally thought to be the main producers of the type 1 IFNs (IFNα and IFNβ) in JDM. This notion may be challenged by a recent study measuring circulating IFNα with a highly sensitive assay and investigating the cellular source of IFNα in several systemic inflammatory diseases. JDM patients had higher levels of circulating IFNα than patients with systemic lupus erythematosus (SLE), but lower levels than patients with monogenic interferonopathies. However, neither isolated circulating pDC nor other circulating immune cell subsets from JDM patients expressed more IFNα than cells from healthy controls, suggesting that a non-circulating cellular source may be responsible for IFNα production in JDM (138).

Due to the lack of available methods to measure circulating IFNα and IFNβ until recently, the type 1 IFN signature, consisting of genes upregulated in response to IFNα or IFNβ stimulation, was used as a surrogate marker of type 1 IFN levels. The type 1 IFN signature in whole blood of three mixed DM and JDM cohorts correlated weakly to moderately with global disease activity [spearman *r* (*r*_s_) = 0.33–0.44] and muscle activity (*r*_s_ = 0.44–0.47), while single IFN signature related serum chemokines MCP-1, IP-10 (CXCL10) and ITAC (CXCL11) had moderate to strong correlations with global (*r*_s_ = 0.42–0.66), muscle (*r*_s_ = 0.44–0.50), and extraskeletal disease activity (*r*_s_ = 0.42–0.55) (115– 117). MxA expression in PBMC, also used as a surrogate for the IFN signature, had a very strong correlation with muscle disease activity of JDM patients (*r*_s_ = 0.80) at disease onset, but not with skin disease activity (89). IFNα activity measured by a functional reporter assay was also higher in JDM patients than controls (90).

Recently, IP-10, TNF receptor 2 (TNFR2) and galectin-9 were found to strongly correlate with global disease activity (*r* = 0.60–0.75) in two studies by Enders et al. (93, 94) IP-10, together with MCP-1 and eotaxin, was also higher in 54 JDM patients a median of 17 years after disease onset than matched healthy controls (92). TNFR2 correlated with CK in a mixed IIM cohort (*r*_s_ = 0.55) (118). Galectin-9 was recently identified as a biomarker for the IFN signature in SLE and anti-phospholipid syndrome (139). IP-10, TNFR2 and galectin-9 are promising biomarkers for disease activity, as they can potently discriminate between active disease and remission even during treatment (93, 94). After stem cell transplantation and concomitant eradication of circulating immune cells, their levels stayed high over several months, which suggests that these proteins are not primarily produced by circulating immune cells, but rather by non-circulating immune or tissue cells, just as IFNα (94, 138). IP-10 and galectin-9 are currently being validated as biomarkers for disease activity in two large international JDM cohorts.

One of the best investigated biomarkers so far in JDM is neopterin, a catabolic product of guanosine triphosphate, which was previously shown to be a marker of immune activation that can be induced by stimulation with IFNγ (140). In the first study identifying serum neopterin as a biomarker for JDM, neopterin levels correlated strongly with muscle strength impairment in 15 JDM patients (*r*_s_ = 0.68) (96). Elevated serum neopterin levels at diagnosis compared to remission were confirmed in an independent cohort (95). In a juvenile myositis validation cohort, plasma neopterin (*n* = 13), and quinolonic acid (*n* = 24), however, did not correlate with myositis disease activity measures (97). Urine neopterin (*n* = 45) moderately correlated with global (*r*_s_ = 0.42), muscle (*r*_s_ = 0.50–0.62) and skin activity (*r*_s_ = 0.49), and edema on MRI (*r*_s_ = 0.55). Urine quinolonic acid also correlated with global and muscle activity and edema on MRI (*r*_s_ = 0.45–0.61) (97). Despite these efforts of validation, neopterin has not been widely implemented into clinical practice as a biomarker for disease activity in JDM.

##### Other inflammatory mediators

Next to type 1 IFN-related markers, other inflammatory mediators have been studied as biomarkers for JDM. The innate TLR4 ligand myeloid related protein 8/14 (MRP8/14 or S100A8/9), originally found to be elevated in patients with systemic-onset juvenile idiopathic arthritis (JIA), correlated moderately to strongly with global and muscle disease activity in a large cohort of 56 JDM patients (*r*_s_ = 0.55–0.65) (98, 141). Another marker adopted from studies in JIA, the soluble IL-2 receptor, was elevated at disease onset compared to remission (95, 142). Serum/plasma levels of the more conventional pro-inflammatory cytokines IL-6, IL-8, and TNFα also moderately correlated with global (*r*_s_ = 0.19–0.46) and muscle disease activity (*r*_s_ = 0.35–0.52) in three mixed JDM and DM cohorts (116, 117). Remarkably, CRP levels did not increase during disease flares (99). BAFF and especially its antagonistic non-cleavable form ΔBAFF, both important for survival and maturation of B cells, moderately correlated with global, muscle and extraskeletal VAS (*r*_s_ = 0.27–0.54), and CK (*r*_s_ = 0.37) in two mixed IIM cohorts (119, 120).

##### Markers related to vasculopathy and cardiovascular risk

Due to the vasculopathic component of JDM, markers related to endothelial activation and dysfunction were explored for their association with disease activity. Von Willebrand factor (vWF) was increased during most periods of active disease in a prospective cohort study, but did not reliably predict disease flares in another study (107, 108). sICAM-1, a marker of endothelial activation, was higher during active disease than remission in a combined cohort of juvenile patients with various systemic autoimmune diseases. VCAM-1, sICAM-3, and L-selectin did not correlate with disease activity, although expression of MiRNA-10a in JDM muscle, which is negatively associated with VCAM-1 expression, showed a correlative trend with muscle and global DAS [Pearson *r* (*r*_p_) = −0.45] (110, 123, 124). C3d and fibrinopeptide A, which are related to vasculopathic changes, were higher in JDM patients with active disease than in remission (109). Endothelial progenitor cell numbers did not differ between JDM patients and controls and did not correlate with disease activity (111).

In view of the increased cardiovascular risk in JDM patients, the lipid profile has been investigated in relation to disease activity (41). Serum HDL negatively correlated with muscle activity (*r*_s_ = −0.54), but not global or skin activity (112). Triglyceride levels correlated strongly with global disease activity assessed by DAS (*r*_s_ = 0.61) and LDL was higher in patients with a higher disease activity (113). Gene expression of the adipokine resistin in PBMC was also upregulated in JDM patients compared to controls and moderately correlated with global and muscle disease activity (*r*_s_ = 0.51 and *r*_s_ = 0.50, respectively) (114). These results indicate that the cardiovascular risk profile is more pronounced in JDM patients with active disease.

##### Circulating immune cell subsets as biomarkers for disease activity

Among the circulating immune cell subsets, T cells and B cells have been studied most extensively in relation to disease activity in JDM. In two mixed cohorts of JDM and DM patients, the frequency of T cells, and especially CD8+ and IFNγ-producing T cells, was decreased during active disease, while the frequency of B cells and IL-4 producing CD4+ T cells was increased compared to remission (121, 122). This may suggest a shifted balance toward a T helper 2 (Th2) type immune response. In cohorts with only JDM patients, total B cell numbers were also increased compared to controls and changes in B cell frequencies accompanied changes in disease activity (*r*_s_ = 0.47) (100, 106). Within the B cell compartment, numbers and frequencies of circulating immature transitional B cells correlated strongly with global disease activity (*r*_s_ = 0.69–0.71). Compared to healthy pediatric controls, these specialized B cells were highly proliferative, had a prominent IFN signature and produced less of their regulatory signature cytokine IL-10 (106). Plasmablast frequencies were also increased during active disease compared to remission (102).

Several T cell subsets have been studied in JDM. In 26 new-onset JDM patients the blood gene expression of Th17-related genes, such as RORC and IL-17F, Th1-related genes, including STAT4, and Th2-related genes, including GATA3 and STAT6, was studied in relation to disease activity. RORC, IL-17F, STAT4, and GATA3 positively correlated with muscle activity and RORC and STAT4 correlated with global activity. This would suggest that the immune response is not specifically skewed toward a certain T helper response. However, at baseline, JDM patients had higher gene expression of Th17 related cytokines IL-23, IL-17F, IL-6, and IL-21 than DM patients, indicating that the Th17 pathway may play a more prominent role in the pathogenesis of JDM than DM. Changes in BCL6, a transcription factor for follicular helper T cells, correlated negatively with a change in extramuscular activity (105). Within CXCR5+ follicular helper T cells, the Th1 subset was decreased in active JDM compared to remission and controls, and Th2 and Th17 subsets were increased in JDM compared to controls (102). Regulatory T cell frequencies in muscle biopsies did not correlate with muscle activity, but suppressive activity of circulating Tregs may be impaired during active disease (104). Finally, global disease activity correlated moderately with the activation status of circulating T cells assessed by CD69 expression (*r*_s_ = 0.43), but not with CD25 and HLA-DR expression (100, 103). The expansion and functional alteration of particular B cell and CD4+ T cell subsets, coinciding with changes in disease activity, hints toward the involvement of these cell subsets in the pathogenesis of JDM.

In conclusion, many circulating, either soluble or cellular, markers have been studied for their relation with muscle and global disease activity. Correlations with disease activity were only moderate for most markers, and some of these molecules are relatively unstable in blood samples or complicated to measure, rendering them unsuited for use in clinical practice. The highest correlations with disease activity were found for markers related to the IFN signature, the lipid profile, for MRP8/14, and immature transitional B cells. However, most of these biomarkers were identified in small patient cohorts and except for neopterin, so far none have been reproduced or thoroughly validated in independent and large JDM cohorts. Neopterin was investigated in a validation cohort, but its correlation with disease activity could only be confirmed in urine, not in plasma. Galectin-9 and IP-10 are currently being validated in two international cohorts and are promising biomarkers for implementation in clinical practice due to their high sensitivity and stability in serum.

#### Biomarkers for Extramuscular Disease Activity

Next to markers for global and muscle disease activity, biomarkers for involvement of specific tissues and organs have been investigated. Four studies by Kobayashi et al. have focused on biomarkers for ILD, and specifically the rapid progressive (RP-ILD) and chronic ILD type, in a Japanese JDM cohort. Not only the presence, but also the level of anti-MDA5 was a sensitive and specific marker for ILD, with the highest levels found in patients with RP-ILD (8, 143, 144). In addition, BAFF, APRIL, KL-6, and IL-18 levels were higher in patients with RP-ILD compared to chronic ILD and JDM patients without ILD (145). KL-6 was prognostic for ILD, as it stayed high in patients with persistent damage on HRCT (144). Biomarkers for cardiac involvement were tested in a Norwegian JDM cohort, a median of 17 years after diagnosis. Eotaxin and MCP-1 were elevated in patients with cardiac dysfunction and correlated moderately to strongly with systolic and diastolic dysfunction especially in patients with persistently active disease (*r*_s_ = 0.45–0.65) (146). In the same cohort, a reduced heart rate variability, which is an indicator of cardiac disease, correlated moderately with ESR, hsCRP, and also MCP-1 and eotaxin levels (*r*_s_ = 0.29–0.47) (147). Next to the autoantibody NXP2, which is prognostic for the development of calcinosis, phosphorylated matrix Gla protein was shown to be higher in patients with calcinosis than without calcinosis (79, 148). Reduced osteocalcin levels were found to be predictive of reduced bone mass, even before start of steroids (149). The presence of the TNFα-308A allele is associated with a more severe disease in JDM. However, apparent associations with this allele are likely to reflect the association with ancestral haplotype 8.1 due to linkage disequilibrium and should be interpreted with this in mind (150). Patients with this genotype are reported to show prolonged symptoms requiring ≥36 months of immunosuppressive therapy, a higher incidence of pathologic calcifications, increased production of TNFα by peripheral blood mononuclear cells *in vitro* and JDM muscle fibers *in vivo*, a higher IFNα activity and a higher rate of complications arising from occlusion of capillaries. Vascular occlusion has been linked to higher levels of the anti-angiogenic thrombospondin-1 (90, 151–154). In summary, a number of potential biomarkers for extramuscular disease activity has been identified, and especially for ILD and cardiac dysfunction the biomarkers seem promising. Validation in independent cohorts will have to confirm their potential as biomarkers for these extramuscular symptoms.

### Histopathology of Muscle and Skin Biopsies

The diagnostic criteria for JDM by Peter and Bohan encompass histopathological findings consistent with DM: “necrosis of myofibers, phagocytosis, regeneration with basophils, large vesicular sarcolemmal nuclei, and prominent nucleoli, atrophy in a perifascicular distribution, variation in fiber size and an inflammatory exudate, often perivascular” (155, 156). For a long time, muscle biopsies were therefore taken as part of routine diagnostic workup. However, with evolving diagnostic options and more specialized trained pediatric rheumatologists muscle biopsies are currently not always considered a necessity for diagnosis (135).

One of the main problems hindering standardized evaluation of muscle biopsies was the lack of an internationally agreed upon scoring tool. An international consensus group of pediatric rheumatologists and pathologists developed such a tool, which encompasses 4 histopathological scoring domains: inflammatory, vascular, muscle fiber and connective tissue changes (157). The scoring tool has now been validated in an independent cohort consisting of 55 patients and was found to correlate with clinical measures of disease activity, including CMAS, PGA, and MMT-8 (*r*_s_ = 0.40–0.62) (45). Muscle biopsy scores may also have prognostic potential: in combination with MSA group, these scores were found to predict the risk of remaining on treatment over time, based on analysis of muscle biopsies from 101 JDM patients (158).

**Figure 1:**
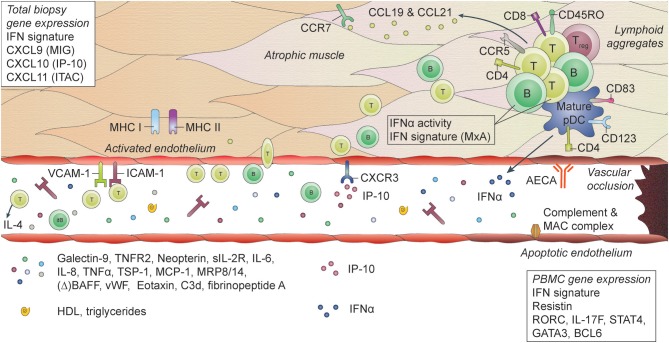
Histopathological features and biomarkers in JDM. JDM is characterized by vasculopathic changes in the tissues, with loss and dysfunction of endothelial cells, leading to capillary dropout and subsequent atrophy of muscle fibers. The exact chain of events leading to loss of blood vessels and muscle fibers is not known, but it is thought that both overexpression of MHC-I (and MHC-II) on myocytes and endothelial damage are early events in the cascade (159, 170). They result in the first attraction of immune cells to the tissue, probably by a stress response of the myocytes and endothelial cells, leading to a first production of chemoattractants. The immune cell infiltrates, which can be organized in lymphoid structures, consist mostly of CD4+ and CD8+ memory T cells, B cells, mature plasmacytoid dendritic cells (pDC) and monocytes. CD4+ and CD8+ T cells are considered responsible for direct killing of muscle cells. pDC are considered the main producers of type I interferons (IFNs), which explains the IFN-I signature that is found in the muscles of JDM patients. Some typical IFN-inducible chemokines, CXCL9 (MIG), CXCL10 (IP-10), and CXCL11 (ITAC), are known for their angiostatic properties. The receptor for these cytokines, CXCR3, is upregulated on endothelial cells in JDM muscle, which may be one of the factors contributing to endothelial dysfunction (137). Other factors include anti-endothelial circulating antibodies (AECA), complement and membrane attack complex (MAC) deposition on endothelial cells. Endothelial cells in muscle also express high levels of ICAM-1 and VCAM-1, which further enables extravasation of immune cells into the tissues and promotes a positive feedback loop resulting in further tissue damage. Not only immune cells in the tissues, but also circulating immune cells show a type I IFN signature and increased IFNα activity. Various circulating markers reflecting immune activation and endothelial activation or distress are increased during active disease in JDM and can potentially be used as biomarkers for disease activity.

The most common findings in muscle biopsy specimens in JDM compared to healthy individuals or patients with non-inflammatory muscle diseases, are profound upregulation of MHC I expression on muscle fibers, increased expression of integrins and complement and membrane attack complex deposition on capillaries and perimysial large vessels, a type 1 IFN signature and immune cell infiltrates consisting mostly of mature pDC, memory CD4+ T cells, and B cells (48, 52, 159–169). (Figure 1) The IFN signature, measured by expression of MxA, correlated with muscle disease activity (166). In skin biopsies similar features are found, with the additional presence of diffuse mast cell infiltration (164).

Several studies have suggested associations between histopathological findings in muscle biopsies and disease duration before the biopsy or disease severity at a later time point. Biopsy specimens taken after a short duration of untreated disease (<2 months), showed higher expression of VCAM-1 (which correlated with higher serum soluble VCAM-1) and expression of genes involved in stress response and protein turnover, whereas biopsies taken after more than 2 months of untreated disease had more pDC infiltration, higher expression of genes involved in the immune response and vascular remodeling and more apoptosis-related markers (171–173). Thus, it should be taken into account that histological findings can depend on the disease duration before the biopsy. In addition, these findings may indicate that endothelial activation is an early feature of JDM, which precedes immune cell infiltration and vasculopathy.

The degree of vasculopathy and vascular injury (as defined by marked capillary dropout, increased direct immunofluorescent arterial staining and lymphocytic vasculitis, amongst others) was associated with a more severe and chronic disease, with severe or persistent weakness, low remission rates at 12 months requiring additional treatment, subcutaneous edema, and chronic ulcerative disease of the skin and gastrointestinal tract (21, 55, 165). The degree of vasculopathy was also correlated with the expression of angiostatic chemokines MIG, IP-10 and ITAC (137). This indicates that the degree of vascular injury may be one of the most important factors determining long-term disease outcomes and that it is related to the IFN signature.

Not only the type of immune cell infiltration, but also the organization of immune cells in the muscle is of significance in JDM. Organization of immune infiltrates in lymphocytic aggregates or lymphoid follicle–like structures with dendritic cells and T cells, as compared to diffuse infiltrates, was associated with a more severe disease course and less response to treatment (174). MHC I expression, one of the most prominent and early histological features in JDM, did not correlate with clinical features of the disease (159, 160, 175).

The importance of thorough and standardized assessment of tissue involvement is underlined by the fact that even in cases with amyopathic DM, with normal EMG and MRI findings, the muscle biopsy can show focal endomysial lymphocyte and macrophage aggregates and 90% positivity for HLA class I in the sarcolemma (176). Unrecognized, low-grade muscle inflammation may be undertreated, resulting in a larger risk of long term damage. However, muscle biopsy is not routinely performed for children with JDM in all centers and therefore in future, biomarkers which are measureable in blood and correlate with biopsy features would represent a major advance.

## Immunopathogenic Implications: Interferons and Vasculopathy

From the biological research conducted in JDM so far, it has become clear that IFNs and their signature play an important role in the immunopathogenesis of JDM (Figure 1). The IFN signature is detectable in muscle fibers, myogenic precursor cells, endothelial cells, skin and several circulating cell subsets of patients with JDM and could point toward a viral etiology (89, 106, 167). Although it has never been demonstrated definitively, several studies suggest that infections may be more common before onset of JDM (177–180). Not only are IFNs potent drivers of (auto)inflammation, they may also be anti-angiogenic factors that could directly or indirectly contribute to endothelial damage and loss in JDM: directly by inhibiting angiogenesis and disrupting the vascular network organization and indirectly by inducing several other angiostatic factors such as galectin-9, IP-10, and ITAC (137, 181–186). In addition, type 1 IFNs inhibit the generation of myotubes and induce atrophy-associated genes in differentiated myotubes. Human skeletal muscle cells can also produce large quantities of IP-10 upon stimulation with IFNγ and TNFα (186, 187).

Rather than being produced by circulating immune cells, IFNs are probably mainly produced within inflamed tissues. Satellite cells, active myogenic cells and endothelial cells in JDM muscle strongly express IFNβ (167). The notion that non-circulating cells within tissues are responsible for IFN production also fits observations by Rodero et al. (138). In particular within muscle of JDM patients the dysbalance between angiogenic and angiostatic factors can contribute to endothelial loss (137, 188). Endothelial cells in JDM muscle downregulate genes related to vessel development, cell adhesion and migration, which are essential for angiogenesis (167). Downregulation of these genes is likely a key event in the development of vasculopathy. Next to being a target of the inflammation, the endothelium may also play an active role in the inflammatory process. In biopsies from JDM patients endothelial cells express inflammatory features, such as high levels of adhesion molecules ICAM-1 and VCAM-1, and produce cytokines and chemokines (161). These can facilitate the attraction and invasion of immune cells into tissues, thereby supporting the inflammatory process and subsequent damage. IP-10 and ITAC were the most highly upregulated genes in endothelial cells from JDM muscle and correlated with the degree of vasculopathy (137, 167). Endothelium-derived IP-10 can even stabilize the interaction between T cells and endothelial cells, thereby possibly contributing to the chronicity of T cell infiltration (189). Recently, a new function has been ascribed to endothelial cells as “semi-professional” antigen presenting cells, which act as sentinels for antigens, and possibly self-antigens, in tissues and facilitate T cell trafficking into these tissues (190, 191). The high expression of MHC molecules on endothelial cells in JDM muscle may support the notion that this process is involved in JDM (160, 175). Although the exact mechanisms of interaction between immune cells and endothelial cells in JDM are still elusive, they may be more elaborate than so far recognized.

## Conclusions and Future Perspectives for Biomarker Research

JDM is a multisystem disease. Not only the skin and skeletal muscles are affected, but also other organ systems and tissues such as the lungs, heart and intestines are frequently (subclinically) involved and may be under-evaluated. Vasculopathy due to loss and dysfunction of endothelial cells as a result of the inflammatory process is thought to underlie the symptoms in most of these organs and tissues. Monitoring of disease activity and damage in all of these affected tissues is important during clinical follow-up, as these are key determinants for the long-term outcomes of patients. Tools for monitoring of tissue activity and damage include histopathological investigation of biopsies, and laboratory testing of blood for specific biomarkers as well as several imaging methods. Each of these methods has their strengths and weaknesses and can be of value for specific diagnostic questions at disease onset or during follow-up, as outlined in the consensus-based recommendations for the management of JDM (135, 192). There is still a need for minimally invasive, but at the same time sensitive and specific diagnostic methods that correlate well with clinical symptoms or reflect low-grade, local inflammation. Tissue-specific biomarkers can therefore be of great value as a monitoring tool.

To be able to identify sensitive, robust and reliable biomarkers or develop monitoring tools, it is of key importance to set up well-defined and large prospective patient cohorts, with a thorough longitudinal collection of a standardized clinical dataset assessing disease activity and organ involvement, paired with collection of patient material (193). Such a dataset is required to ensure a strict definition of active and inactive disease [e.g., as proposed by Almeida et al. (72)]. An important consideration for a successful biomarker study is the timing of data and sample collection: depending on the purpose of the biomarker, time points before start of immunosuppressive treatment, before each adjustment of medication, during flares, at paired time points during active and inactive disease or even at regular intervals of max 3–4 months may be crucial to reliably investigate the potency of a biomarker.

Next to the “classical” statistical approach, comparing patients with active disease and patients in remission (cross-sectionally or in paired samples), new computational approaches providing analysis methods that can integrate longitudinal data from multiple patients and multiple (bio)markers or scoring tools should be considered. These methods take into account the fluctuating nature of a relapsing-remitting disease such as JDM and are therefore better suited to test the reliability of a tool that will be used for longitudinal follow-up in clinical practice (194, 195).

To achieve implementation of a marker or tool into clinical practice, both clinical and technical validation in independent cohorts is of utmost importance. Only few markers prove to be stable, reliable and easy to measure, which are key features for a marker or tool to be suited for implementation into clinical practice. Also the invasiveness of the method should be taken into account. Ideally, a period of experimental implementation can demonstrate the added value and feasibility of a marker or tool in clinical practice. To achieve all this in a large group of JDM patients to ensure sufficient statistical power, international networks with well-established collaborations are fundamental.

Eventually, monitoring of disease activity with a reliable tool can be used to guide treatment and thereby facilitate precision medicine, with high dose therapy when indicated but also preventing overtreatment. This may reduce both the duration of active disease and thereby the disease-inflicted damage, and medication side effects, which will benefit the long-term outcomes on various domains, such as muscle weakness, organ damage, cardiopulmonary fitness, and quality of life. Next to facilitating personalized treatment strategies, newly identified biomarkers may also provide insights into the immunopathogenesis of JDM and provide new treatment targets. For instance, new treatment strategies targeting the IFN signature, such as anti-IFN antibodies (sifalimumab) or JAK-inhibition (ruxolitinib) have been shown to reduce the IFN signature in blood and muscle of adult dermatomyositis patients, and may therefore be promising new strategies for patients with JDM (186, 196, 197). Several studies discussed in this review suggest a strong link between the IFN signature and vasculopathy; and vasculopathy has been related to disease severity. Targeting the IFN signature may thus benefit vascularization in JDM and thereby improve outcomes.

## Author Contributions

JW collected literature and wrote the manuscript draft. FvW and AvR-K supervised JW, outlined the manuscript focus and revised the manuscript. CD and LW critically revised the manuscript.

### Conflict of Interest Statement

The authors declare that the research was conducted in the absence of any commercial or financial relationships that could be construed as a potential conflict of interest.
